# Semiautomatic volume measure of kidney vascular territories on CT angiography to plan aortic aneurysm repair in patients with horseshoe kidney

**DOI:** 10.1186/s41747-024-00531-4

**Published:** 2024-12-02

**Authors:** Axel Bartoli, Alberto Colombo, Francesco Pisu, Tommaso Galliena, Chiara Gnasso, Enrico Rinaldi, Germano Melissano, Anna Palmisano, Antonio Esposito

**Affiliations:** 1 Experimental Imaging Center, IRCCS San Raffaele Scientific Institute, Milano, Italy; 2 Department of Radiology, Hôpital de la TIMONE, AP-HM, Marseille, France; 3 Aix Marseille Univ, CNRS, CRMBM, Marseille, France; 4 School of Medicine, Vita—Salute San Raffaele University, Milan, Italy; 5 Division of Vascular Surgery, IRCCS San Raffaele Scientific Institute, Vita-Salute San Raffaele University, Milan, Italy

**Keywords:** Aortic aneurysm (abdominal), Computed tomography angiography, Fused kidney, Preoperative care, Radiology

## Abstract

**Abstract:**

Surgical repair of abdominal aortic aneurism (AAA) with horseshoe kidney (HK) is challenging because of several accessory renal arteries (RAs), variable in number, branches, and vascular territories, with subsequent variable renal damage. The identification of RAs and vascular territories could contribute to surgical planning. We developed a semiautomatic presurgical computed tomography angiography (CTA)-based model to measure the renal volume of each RA, validated on postsurgical CTA in patients with HK treated for AAA. Renal parenchyma volume was extracted on both CTAs (Vol_Tot_pre_ and Vol_Tot_post_) after labeling RAs ostia and vascular endpoints by two observers using a semiautomatic model by assigning each renal voxel to the closest vascular ending, obtaining volumes for each vascular territory. Number of RAs number was 4.0 ± 1.4 (mean ± standard deviation (SD)), Vol_Tot_pre_ 360 ± 76.5 cm^3^; kidney volume loss at surgery (KVLS) (Vol_Tot_pre_ *minus* Vol_Tot_post_) 51.9 ± 35.4 cm^3^; percentage of kidney loss 15.2 ± 11.6%. KVLS and predicted kidney volume loss on preoperative CTA (PKVL) were strongly correlated (*r* = 0.93; *p* = 0.023). Interobserver agreement was good (mean bias = 0.000001 ± 1.96 SD of 19.1 cm^3^). Presurgical semiautomatic segmentation of vascular territories in patients with HK and AAA is feasible.

**Relevance statement:**

This software allowed the preoperative calculation of renal volume perfused by each renal artery in the challenging association of the horseshoe kidney and abdominal aortic aneurism. It helps to determine the feasibility of surgical resection of arteries, thereby improving surgical planning and reducing the risk of postoperative renal function deterioration.

**Key Points:**

- The association between horseshoe kidney and abdominal aortic aneurism is a challenging condition that may require renal vascular resection.
- A semiautomatic model measures renal volume perfused by each artery on preoperative computed tomography angiography with high accuracy.
- Customized use of this tool could improve surgical management by determining which arteries can be safely resected during surgery.

**Graphical Abstract:**

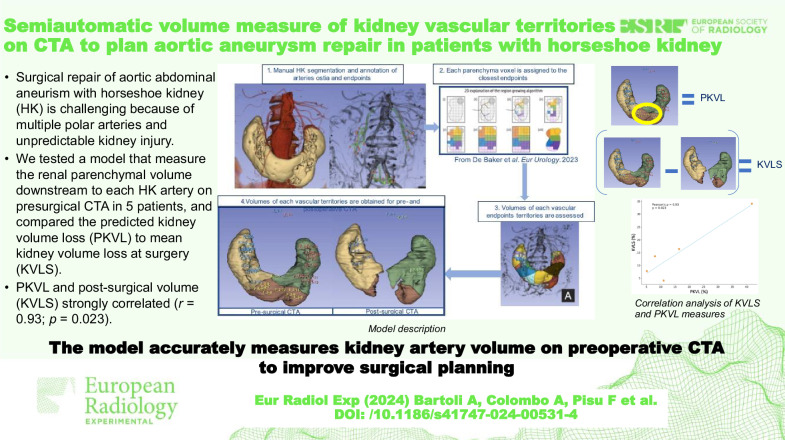

## Background

The association of abdominal aortic aneurysm (AAA) and horseshoe kidney (HK) is a rare and challenging condition that occurs in less than 0.2% of all cases of AAA [1]. The HK is a congenital anomaly in which the kidneys are fused in front of the anterior aortic wall in its most common form. Usually, several polar arteries arise from the distal abdominal aorta or the iliac arteries to perfuse the HK. When an AAA is associated with HK, open surgery is still widely practiced in many centers with a transperitoneal or retroperitoneal approach [2].

A key consideration of the surgery is whether to preserve the variant kidney vascularization or not [3]. Preserving them can be challenging, but their removal can lead to significant renal volume reduction and subsequent loss of function [4, 5]. In open surgery repair, it is recommended that as many arteries as possible should be reanastomosed to the prosthesis, even if it brings additional technical issues, especially in the emergency setting [1, 6].

Computed tomography angiography (CTA) is the best diagnostic test to perform a preoperative vascular assessment [1, 7]. Therefore, it is of interest to develop a method to preoperatively assess the parenchyma volume perfused by each artery to establish whether they should be preserved or not. Recently, de Backer et al [8] developed an algorithm that can predict patient renal tumor perfusion information based on preoperative CTA. There was a high accuracy with preoperative evaluation using a visual method, but no volume measurements were provided. Similarly, Xu et al [9] developed a realistic renal vasculature model based on micro-computed tomography (CT) imaging in rats.

Hence, the purpose of the present study was to develop an experimental semiautomatic model to measure renal volume downstream of each HK artery on presurgical CTA.

## Methods

This study was a retrospective single-center analysis (IRCCS San Raffaele Hospital, Milan, Italy) approved by the Ethics Committee, and patient consent was waived.

All consecutive patients surgically treated for AAA with HK at our institution between January 2019 and December 2023, with adequate pre- and postoperative CTA, were enrolled. All patients had a four-phase CTA acquired on dual source scanner (SOMATOM Definition Flash, Siemens Healthineers, Erlangen, Germany) with high-pitch helical acquisition (120 kVp; current 180–372 mA; slice thickness 1 mm) during biphasic injection of iodinated contrast media (Visipaque320, GE Healthcare, Little Chalfont, Buckinghamshire, UK) at high flow rate (5 mL/s). Contrast bolus volume was tailored to patients’ size: 85 mL for body mass index < 25; 95 mL for body mass index 25–30; and 110 mL for body mass index > 30.

Renal parenchyma was manually segmented with 3D Slicer (https://www.slicer.org, 2014) [10], using the standard “Segment Editor” extension. First, the fill-between-slices tool was initialized and set to auto-update. Contours of the parenchyma were manually drawn every 10 slices, interpolated, and manually refined when necessary. Segmentation was performed on the arterial phase for both preoperative and postoperative CTA to obtain total HK volume in cm^3^: Vol_Tot_pre_ and Vol_Tot_post_, respectively. Renal cysts, caliceal systems, proximal vessels, and fat were excluded from the segmentation. Two radiologists (Reader 1, A.B., and Reader 2, C.G., both with 7 years of experience in abdominal imaging) independently annotated the arterial tree by labeling ostia of all HK arteries and all the corresponding vascular endings that were visible in the parenchyma, blinded to postsurgical CTA. Segmentation from Reader 1 was considered as the standard of reference.

**Fig. 1:**
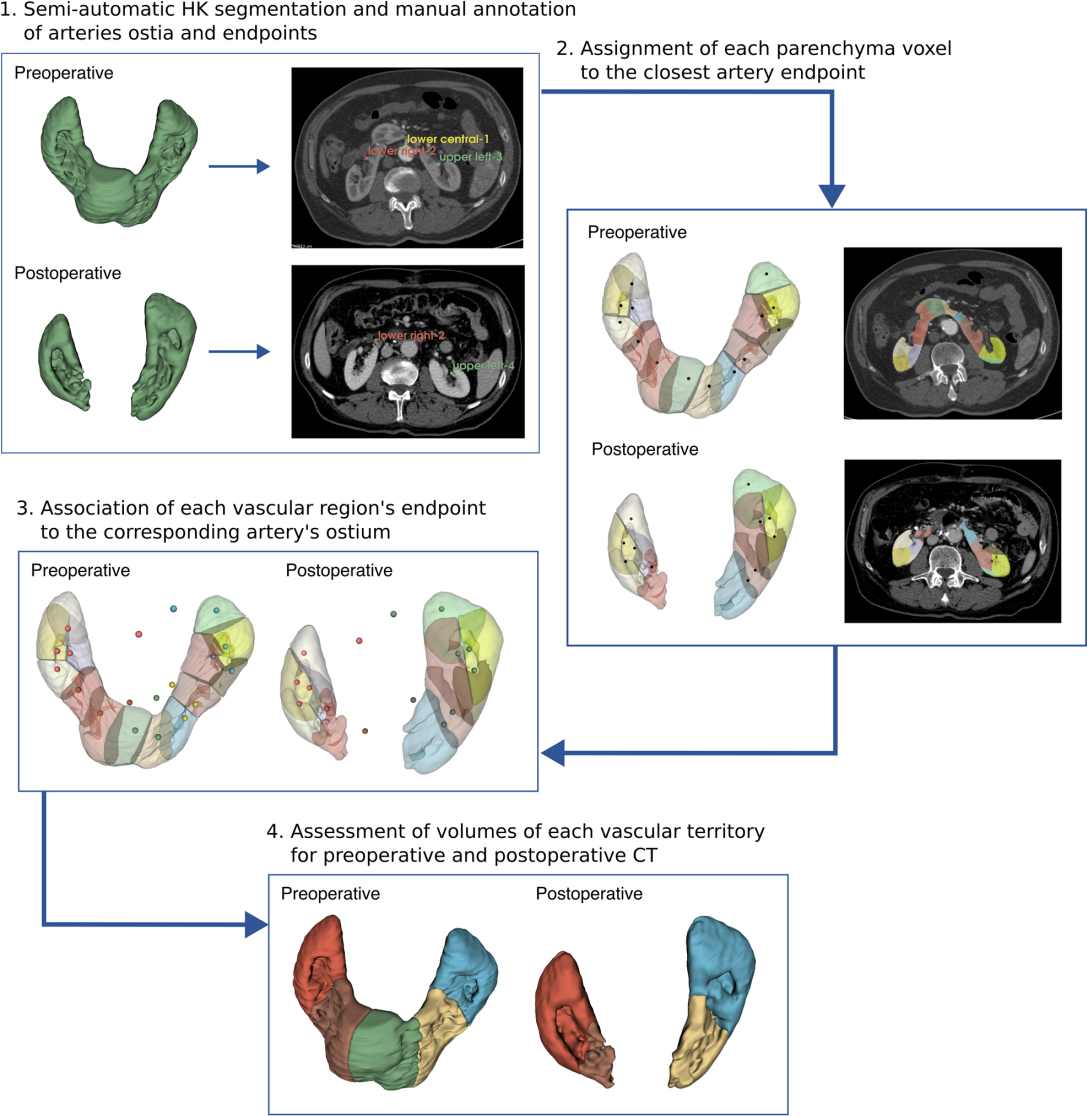
Pipeline description in four steps. CTA, Computed tomography angiography; HK, Horseshoe kidney

The semiautomatic algorithm processes these fiducial landmarks to assign each voxel in the kidney segmentation to its nearest vascular endpoint based on Euclidean distance. This assignment is performed by calculating distance maps between all segmented kidney voxels and the marked vascular endpoints and then determining the closest endpoint for each voxel. This approach effectively divides the kidney volume into regions, each associated with a specific vascular endpoint. The method then computes the volume of each vascular territory by summing the volumes of all assigned voxels, using the voxel dimensions from the CT scan metadata. The algorithm generates three-dimensional assignment maps visualizing the voxel-to-endpoint associations and produces statistical summaries including absolute and relative volumes for each vascular and arterial territory (Fig. 1). The effective renal volume loss, named kidney volume loss at surgery (KVLS) was obtained by subtracting the total postoperative renal volume on postoperative CTA from the total renal volume of the preoperative CTA (KVLS = Vol_Tot_pre_ *minus* Vol_Tot_post_, cm^3^). The predicted kidney volume loss (PKVL) was obtained by subtracting preoperative kidney volume from the volume of parenchyma with vascular endpoints downstream to vessels planned to be closed during surgery. Both KVLS and PKVL were presented as volumes and percentages.

A Bland-Altman interobserver agreement analysis was conducted on the preoperative measures of the renal volumes for each renal artery. A Pearson correlation analysis was performed. Values of *p* < 0.05 were deemed statistically significant. All analyses were performed using R software (version 4.1.0) and Python language (version 3.9).

## Results

A total of five patients with concomitant AAA and HK were included (Supplemental Table S1). Three patients had a Type 3 HK according to Graves classification [11]. AAA maximum diameter was 64 ± 14.8 mm (mean ± standard deviation (SD)). All patients underwent open surgical repair with aneurysmectomy and aortic graft replacement, and isthmus division consisting of partial parenchyma resection of the central part of the HK. The mean ± SD number of renal arteries was 4.0 ± 1.4. The number of renal artery ostia was the same in preoperative and postoperative CTA, except for one patient in which one ostium was no longer recognizable for ligation. In patient #2, a renal artery that arose from the anterior wall of the abdominal aorta was reimplanted to the graft.

**Fig. 2:**
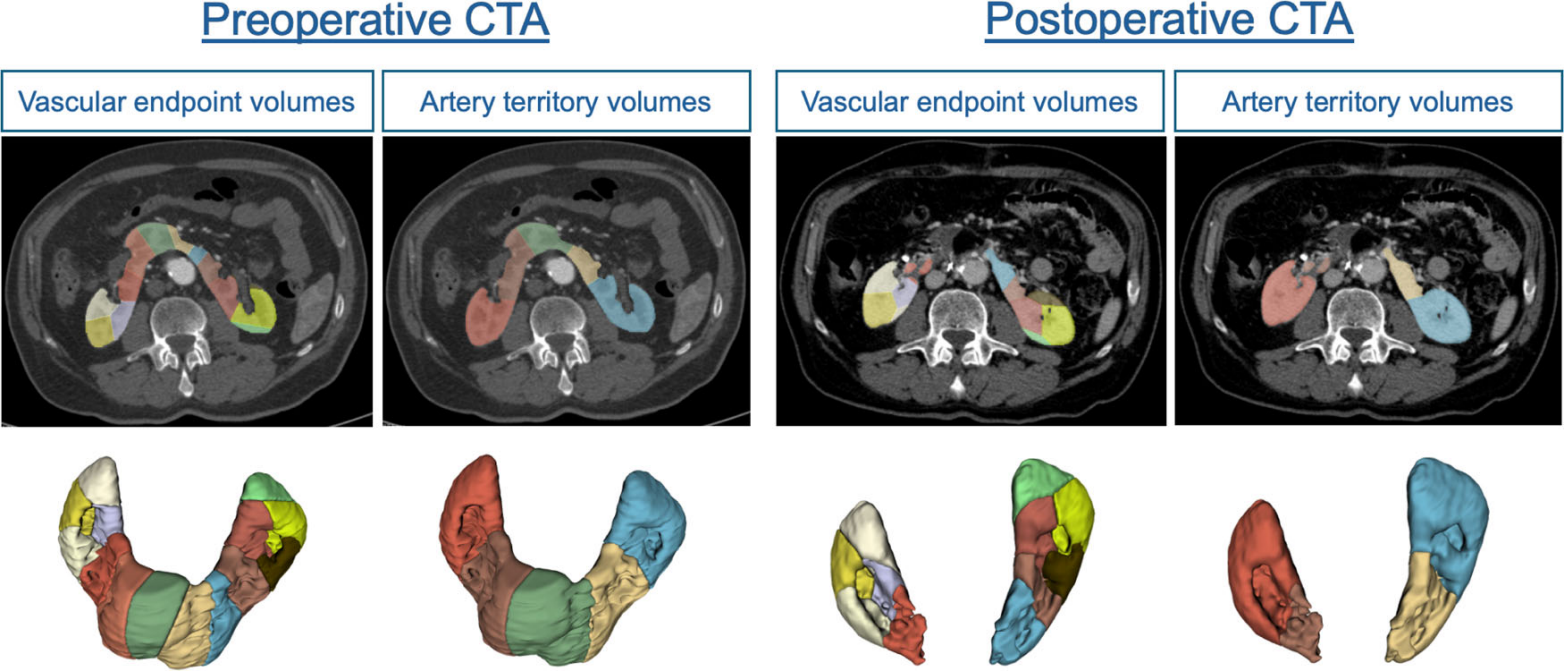
Visual results of the obtained segmentation for both preoperative and postoperative CTA in patient 3. CTA, Computed tomography angiography

An exemplifying case of vascular territories obtained with the semiautomatic segmentation is reported in Fig. 2. All measures obtained after CTA analysis are presented in Table 1. Vol_Tot_pre_ was 360 ± 76.5 cm^3^, and mean KVLS was 51.94 ± 35.4 cm^3^ representing 15.2 ± 11.6% of preoperative volume (Vol_Tot_pre_). Patient #4, who had undergone emergency surgery for a ruptured aneurysm, had a KVLS of 108 (34.1%) cm^3^, which was significantly higher than that of the other participants.

**Table 1:** CTA analysis results

Patient	CTA	Days between surgery and CTA	Arteries ostia (*n*)	HK volume (cm^3^)	KVLS (cm^3^)	KVLS (%)	Vascular endpoints (*n*)	PKVL (cm^3^)	PKVL (%)
1	Presurgical	132	5	447.2	−	−	23	−	−
	Postsurgical	41	5	412.4	34.8	7.8	21	23.0	5.1
2	Presurgical	2	3	331.7	−	−	28	−	−
	Postsurgical	55	3	318.1	13.6	4.1	26	36.5	11.0
3	Presurgical	50	5	271.2	−	−	19	−	−
	Postsurgical	33	4	226.7	44.5	16.4	15	44.5	16.4
4	Presurgical	0	2	317.2	−	−	17	−	−
	Postsurgical	44	2	209.1	108.1	34.1	14	132.7	41.8
5	Presurgical	5	5	432.5	−	−	25	−	−
	Postsurgical	154	5	373.8	58.7	13.6	23	34.4	8.0

*CTA* Computed tomography angiography, *KVLS* Kidney volume loss at surgery, *PKVL* Predicted kidney volume loss

**Fig. 3:**
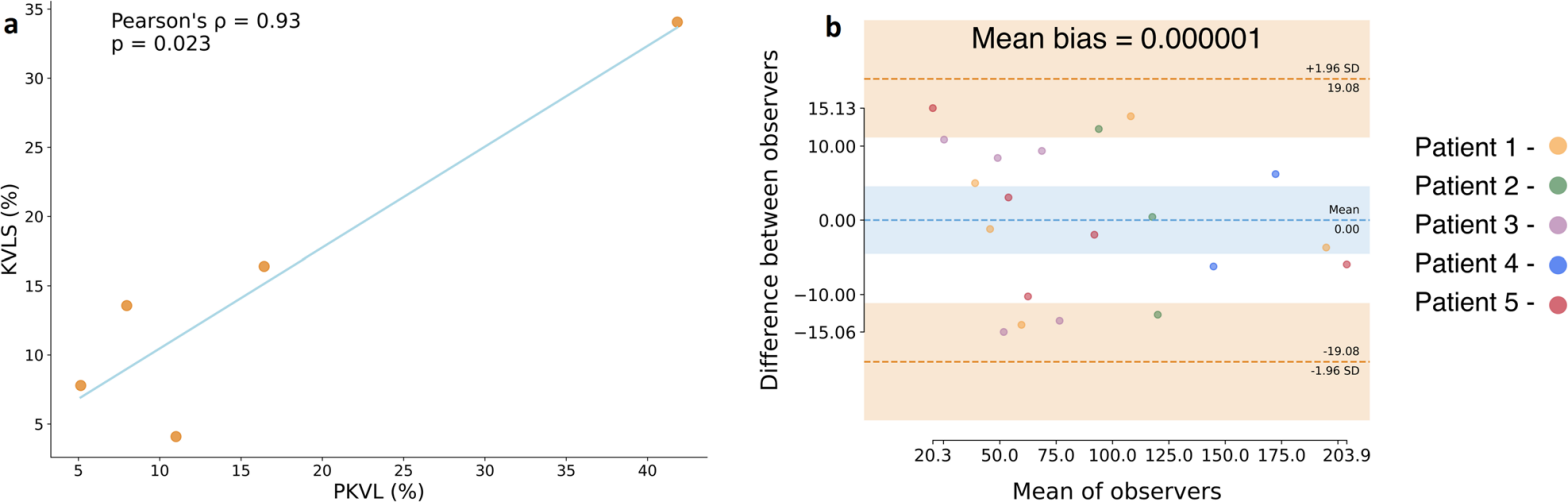
Correlation analysis and Bland-Altman plots. **a** Correlation of KVLS and PKVL measures for the five patients. The blue line is the fitted regression line. **b** Bland-Altman analysis for the artery volume measures between Reader 1 and Reader 2 on the five preoperative CTAs. Blue dashed lines represent the mean differences (bias), and the two orange dashed lines denote ± 1.96 standard deviations from the mean. CTA, Computed tomography angiography; KVLS, Kidney volume loss on surgery; PKVL, Predicted kidney volume loss

Mean PKVL was 54.2 ± 44.5 cm^3^ representing 16.5 ± 14.8% of Vol_Tot_pre_. The percentage of PKVL and KVLS strongly correlated (*r* = 0.93; *p* = 0.023) (Fig. 3a). Bland-Altman plots for all arterial territories volumes on preoperative CT between readers are presented on Fig. 3b. The analysis showed good agreement with a mean difference bias of 0.000001 and a ± 1.96 SD from the mean of 19.08 cm^3^, which represents approximately 5.3% of mean Vol_Tot_pre_.

## Discussion

To the best of our knowledge, the presented method is the first one dedicated to the preoperative measure of the vascular territories volume in HK. This proof-of-concept approach addresses a specific clinical need that is rare but complex. It allows each HK vascular volumes to be measured on the preoperative CTA, guiding surgical planning. This ready-to-use tool provides a specific answer to a clinical problem and could be shared with teams doing this type of intervention on a case-by-case basis.

Each renal artery, in its classic anatomical pattern, divides into two main trunks, with most often five to seven terminal arteries (ranging from 2 to 10) with no intrarenal anastomoses, which supply individual segments of the kidney. Polar arteries are not auxiliary or accessory but participate in the normal segmental branches. The atypical origin of the renal arteries is quite common in normally positioned kidneys and extremely common in ectopic kidneys and HK. In a common nonfused kidney, two renal arteries per kidney are found in 22% of the population, and three or more renal arteries in 4% of the population, with different patterns. In HK, vascular variants are common, and an atypical origin of polar renal arteries may be found in up to a quarter of the population [12].

Therefore, an objective method to evaluate the volume of kidneys supplied by each individual polar artery could become a valuable method in the surgical decision-making process. During endovascular treatment of aortic aneurysm, polar arteries may be technically reattached to the main endograft by means of fenestrations or branches. However, arteries below 4 mm in diameter, even if reattached, have a very low chance of staying patent and are often sacrificed primarily [13]. In this instance, if the volume of the kidney supplied is clinically relevant, the decision could lean towards open repair, where surgical reimplantation of the polar arteries is almost always technically possible, either directly or with an interposition graft.

We found a strong correlation between the PKVL and the KVLS with good interobserver reproducibility. One of the aspects of our analysis, and the difficulty of comparing it with other studies, is that it cannot be based on an artery level but on an endpoint-level analysis. In fact, for 4 out of 5 patients, no artery was ligated, but a relevant percentage of the fused kidney was removed. Hence, this surgical section does not always follow a vascular frontier, so there were no clear boundaries. We assumed that when an endpoint was no longer visible, the entire associated volume was removed. However, the boundary was blurred, and we know that adjacent territories can participate in the revascularization of these territories, explaining some of the differences between PKVL and KVLS.

This method has the advantage of being independent of the surgical approach used. On the other hand, it is affected by the capability to recognize vascular endpoints, therefore its estimation of the PKVL can be influenced by the slice thickness of scan reconstruction. A method for measuring the central portion volume of the HK, based on watershed lines, has been proposed by Handa et al [14]. For one single case reported in this study, the isthmic volume was 24% of the total volume, a rate which is consistent with our mean KVLS measure of 15.2 ± 11.6%. However, this sign is not systematically present, and relying solely on its analysis is not sufficient. A previous study using selective accessory artery angiography with spiral CT showed that isthmic renal arteries supplying less than 32% of the total parenchyma could be occluded by an endovascular graft without renal dysfunction [4].

In our study, one patient had a KVLS of 34%. In their study, Fabiani et al [15] included 9 patients who had endovascular aortic repair with coverage of the isthmus arteries. One of the patients had two isthmus arteries covered and experienced postoperative renal failure that required 3 weeks of hemodialysis. Considering that some patients may already have underlying renal dysfunction (and that renal protection is recommended for this type of surgery), this report confirms the need to preoperatively know the renal volume associated with each renal artery.

The literature concerning this association consists mainly of case reports and of retrospective series [16–18]. Our population appears to be in line with previously published data. In a retrospective analysis, Majos et al [19] evaluated 83 patients with HK with a mean number of renal arteries of 4.6 ± 1.4 while it was 4.0 ± 1.4 in our population. Patients in our cohort mostly had type 3 Graves classification, which is one of the most frequent forms [11].

Our study has limitations, mainly related to the small number of patients. According to Whanainen et al [1], HK associated with AAA occurs in only 0.12% of AAA cases, from which we must subtract those who will not undergo surgery. Gathering a large cohort about this association is difficult and would require a long-term multicenter study. Of note, the largest recent cohort included 10 patients [20]. Also, our analysis did not integrate the potential stenosis on the vascular network that could affect downstream renal function.

Future works should first focus on the complete automation of Ostia and vascular endings editing. Realistic full-scale models of renal vasculature exist but are built from micro-CT with a high spatial resolution, which is unfeasible in clinical practice [9]. Ultrahigh-resolution photon-counting CT may further improve vascular detection and automatic extraction. Also, future studies correlating the volume loss with glomerular filtration at scintigraphy would be of interest [21].

In conclusion, we proposed a semiautomatic model that can measure vascular territories downstream to each HK artery before AAA. This method allows precise surgical planning with good interobserver agreement, resulting in a powerful method in the multidisciplinary management of these complex cases.

## Supplementary information

**Additional file 1:** **Table S1.** Baseline population characteristics

## Data Availability

The data will be provided by the corresponding author after a reasonable request.

## Abbreviations

AAA: Abdominal aortic aneurysm
CT: Computed tomography
CTA: CT angiography
HK: Horseshoe kidney
KVLS: Kidney volume loss at surgery
PKVL: Predicted kidney volume loss
SD: Standard deviation
