# Supplementary material for: Semiautomatic volume measure of kidney vascular territories on CT angiography to plan aortic aneurysm repair in patients with horseshoe kidney
Source: Eur Radiol Exp. 2024 Dec 2;8:133. doi: 10.1186/s41747-024-00531-4 (PMC11612044; doi:10.1186/s41747-024-00531-4)
Supplement: Supplementary file 1 — Additional file 1: Table S1. Baseline population characteristics [file 41747_2024_531_MOESM1_ESM.pdf]

Semiautomatic volume measure of kidney vascular territories on CT angiography to plan aortic aneurysm repair in patients with horseshoe kidney

ELECTRONIC SUPPLEMENTARY MATERIAL

Table S1 – Baseline population characteristics

Patient	Age	Sex	Surgery	Emergency	AAA diameter (mm)	HK Classification	Pre - surgery GFR (mL/min)	Post-surgery GFR (mL/min)
1	74	M	Open	No	50	Type 3	105.1	106.7
2	64	M	Open	No	69	Type 2	78.2	84.4
3	79	M	Open	No	61	Type 3	86.3	90.1
4	64	M	Open	Yes	87	Type 1	70.6	51.4
5	68	M	Open	No	53	Type 3	60.5	41.0

Note - AAA abdominal aortic aneurysm, GFR glomerular filtration rate, HK horseshoe kidney.
